# A Rare Coronary Anomaly: One Ostium Fits All

**DOI:** 10.4061/2010/476760

**Published:** 2010-07-20

**Authors:** C. Mihl, B. S. N. Alzand, M. H. Winkens

**Affiliations:** ^1^Department of Cardiology, Maastricht University Medical Center, P.O. Box 5800, 6202 AZ Maastricht, The Netherlands; ^2^Department of Cardiology, Canisius Wilhelmina Hospital Nijmegen, P.O. Box 9015, 6500 GS Nijmegen, The Netherlands

## Abstract

Coronary anomalies affect a small percentage of the general population. A solitary coronary ostium in the absence of other major congenital anomalies is very rare. We describe a case of a patient, admitted to our cardiology department with an acute myocardial infarction. A coronary angiogram shows a solitary ostium originating from the right sinus of Valsalva with the left anterior descending coronary artery (LAD) ventral to the pulmonary artery and the circumflex artery (Cx) following its course retroaortically. The theoretical variant of this type of malformation has been described but has not been reported in a clinical case before. Coronary anomalies are usually detected during coronary angiography, but exact course determination and relationships are difficult to visualize. The use of cardiac computed tomography (CCT) allows visualization of the coronary anatomy in a 3-dimensional image and demonstrated an added value to coronary angiography.

## 1. Case Report

**Figure 1:**
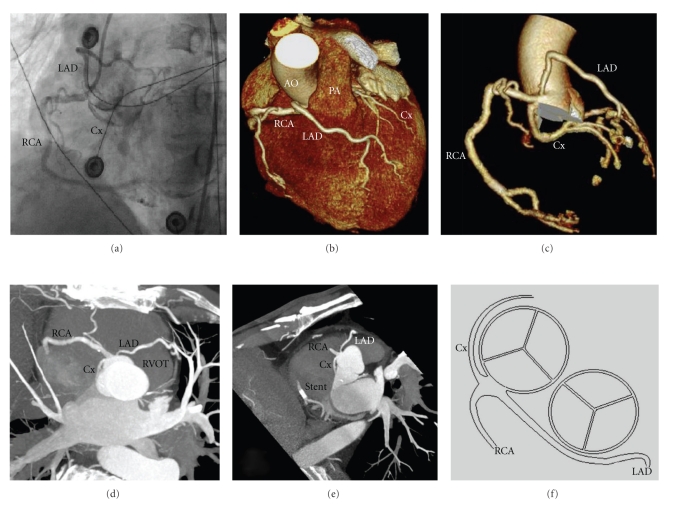
(a) Conventional coronary angiogram in left anterior oblique projection picturing three coronary arteries originating from the right sinus of Valsalva. (b) Coloured volume rendering showing the LAD crossing ventral to the pulmonary artery. (c) A three-dimensional reconstruction showing 3 coronary arteries arising from the right sinus of Valsalva. (d) and (e) Contrast-enhanced 64-slice CT coronary angiography showing a solitary coronary ostium with a visible stent in the distal segment of the RCA. (f) Schematic drawing of the coronary anomaly.

A 69-year-old male was admitted to our cardiology department because of sudden onset retrosternal pain. His past medical history was unremarkable, and he never had anginal complaints before. His electrocardiogram showed ST-elevations in the inferior and lateral leads and ST-depression over the precordial leads consistent with inferoposterolateral wall myocardial infarction. A coronary angiogram was performed immediately and showed a subtotal occlusion of the distal right coronary artery (RCA) in which a bare metal stent was placed. Moreover, the RCA, the left anterior descending coronary artery (LAD), and the circumflex artery (Cx) were all originating from a solitary ostium from the right sinus of Valsalva (Figure 1(a)). The path of the LAD is ventral to the pulmonary artery and is not coursing between the pulmonary artery and the aorta. ECG-gated 64-row multislice cardiac computed tomography (CCT) confirmed the course of the coronaries and showed a solitary coronary ostium (Figures 1(d) and 1(e)) with a retroaortic course of the CX (Figures 1(c), 1(d), and 1(e)), the LAD runs ventral to the pulmonary trunk (Figures 1(b), 1(d), and 1(e)), and the RCA courses normally with a stent visible in segment 3 (Figures 1(b), 1(c), and 1(e)). Medical therapy was prescribed and patient was discharged without symptoms. 

## 2. Discussion

According to the literature, coronary anomalies affect 0.3–5.6 percent of the general population [1]. This variable percentage is derived from angiographic and necropsy studies which are influenced by entry biases and unclear criteria. The incidence of coronary anomalies is clinically relevant in view of the fact that they are the cause of 19% of sudden death in athletes [2]. A solitary coronary ostium in the absence of other major congenital anomalies is very rare with an incidence ranging from 0.019 to 0.4% [3–6]. Anomalous coronary arteries are associated with ischemia and sudden death, which could be the result of compression by the aorta and pulmonary artery. However, ischemia is also reported when an anomalous coronary artery does not run between the great vessels [7]. Our patient has a solitary ostium originating from the right sinus of Valsalva with the LAD ventral to the pulmonary artery and the Cx following its course retroaortically, a variant which has never been reported before. Coronary anomalies are usually detected during coronary angiography, but exact course determination and relationships are difficult to visualize. The use of CCT allows visualization of the entire course of the coronary artery in a 3-dimensional image. 

## Disclosures

All authors have read and approved the paper and no part of this paper is being published or under consideration for publication elsewhere. There are no conflicts of interest for any of the authors. 

## References

[1] Angelini P, Velasco JA, Flamm S (2002). Coronary anomalies: incidence, pathophysiology, and clinical relevance. *Circulation*.

[2] Maron BJ, Thompson PD, Puffer JC (1996). Cardiovascular preparticipation screening of competitive athletes: a statement for health professionals from the Sudden Death Committee (Clinical cardiology) and Congenital Cardiac Defects Committee (Cardiovascular disease in the young), American Heart Association. *Circulation*.

[3] Yamanaka O, Hobbs RE (1990). Coronary artery anomalies in 126 595 patients undergoing coronary anteriography. *Catheterization and Cardiovascular Diagnosis*.

[4] Desmet W, Vanhaecke J, Vrolix M (1992). Isolated single coronary artery: a review of 50 000 consecutive coronary angiographies. *European Heart Journal*.

[5] Lipton MJ, Barry WH, Obrez I (1979). Isolated single coronary artery: diagnosis, angiographic classification, and clinical significance. *Radiology*.

[6] Shirani J, Roberts WC (1993). Solitary coronary ostium in the aorta in the absence of other major congenital cardiovascular anomalies. *Journal of the American College of Cardiology*.

[7] Sayar N, Terzi S, Akbulut T (2005). Single coronary artery with subsequent coursing of right coronary artery between aorta and pulmonary artery: fractional flow reserve of the anomalous artery guiding the treatment. *International Heart Journal*.

